# Milk Phospholipid Profiling Among Japanese Women with Differing Docosahexaenoic Acid Levels

**DOI:** 10.1097/PG9.0000000000000058

**Published:** 2021-03-30

**Authors:** Hiroshi M. Ueno, Andrew MacKenzie, Dawn Scott, Satoshi Higurashi, Yasuhiro Toba, Toshiya Kobayashi

**Affiliations:** From the * Research and Development Department, Bean Stalk Snow Co., Ltd., Kawagoe, Japan; † Callaghan Innovation, Wellington, New Zealand.

**Keywords:** polar lipids, phosphorus-31 nuclear magnetic resonance, human milk

## Abstract

To characterize the milk phospholipids (PLs) profile using phosphorus-31 nuclear magnetic resonance (^31^P-NMR) and to investigate the effect of elevated milk docosahexaenoic acid (DHA) levels on PLs profiles in Japanese mothers.

**Methods:**

Milk samples from eligible patients with high and low DHA from a former cross-sectional study (n = 20; n = 10 for each group) were included. Fifteen milk PLs were analyzed using ^31^P-NMR, and the profiles were compared group-wise using Mann–Whitney *U*-test. The *P* value of <0.05 was considered statistically significant.

**Results:**

The median DHA content in milk was 1.13% and 0.29% for the high and low milk DHA groups, respectively. Twelve PLs, excluding lysophosphatidylserine, cardiolipin, and phosphatidylglycerol, were detected in all participants with 100% positive results. The median concentrations and proportions of total PLs, sphingophospholipids, and glycerophospholipids were comparable between groups. The proportions of choline-containing glycerophospholipid were significantly higher in the high milk DHA group than that in the low milk DHA group (24.09% [median, interquartile range: 23.08%–26.38%] and 21.41% [20.74%–22.84%], *P* = 0.019). Although the proportions of phosphatidylinositol were significantly lower in the high milk DHA group than that in the low milk DHA group (6.62% [5.75%–6.72%] versus 7.63% [7.11%–8.16%], *P* = 0.002), while that of phosphatidylcholine (21.90% [18.51%–23.22%] versus 19.78% [18.17%–20.26%], *P* = 0.059) and alkyl-acyl phosphatidylcholine (0.60% [0.40%–0.74%] versus 0.33% [0.14%–0.51%], *P* = 0.059) were higher in the former than that in the latter.

**Conclusions:**

Our results were comparable to that of the previous literature. Large variations in the milk DHA might affect the composition of choline-containing glycerophospholipids in Japanese mothers. However, possible confounders were not excluded in the study populations.

## Abstract 2

Supplemental Digital Content is available in the text.

**What Is Known**

- Docosahexaenoic acid (DHA) and phospholipids (PL) are important for neurological development in infants.
- Milk DHA levels are influenced by maternal characteristics including ethnicity, diet, genetics, and socioeconomic factors.

**What Is New**

- Twelve of 15 subclasses of PLs (excluding lysophosphatidylserine, cardiolipin, and phosphatidylglycerol) were determined using 31-phosphorus nuclear magnetic resonance spectroscopy in all participants with 100% positive results.
- The milk PL profiles corroborated previous reports, including minor subclasses such as plasmalogen, phosphatidic acid, phosphatidylglycerol, and cardiolipin.
- The composition of choline-containing glycerophospholipids appeared to be different among mothers who had high dietary and milk DHA.

## INTRODUCTION

Milk fat globule membranes are involved in the development of cognitive and gastrointestinal functions during early-life nutrition (1). Phospholipids (PLs) are a class of polar and phosphorus-containing lipids present in membrane-associated constituents. Such major constituents include phosphatidylcholine (PC), sphingomyelin (SM), phosphatidylethanolamine (PE), phosphatidylserine (PS), and phosphatidylinositol (PI) (2), whereas minor constituents include Lyso-PLs, phosphatidic acid, phosphatidylglycerol, plasmalogens, and cardiolipin (3,4).

Globally, the mean concentration of docosahexaenoic acid (C22:6n-3, DHA) in the human milk has been calculated to be 0.3%; however, this figure varies widely according to the geography and local environmental factors (5). In Japan, the mean milk DHA concentration ranges from 0.6% to 1.1% and is affected by the consumption of seafood and DHA supplements (6–9). Fifteen percent of long-chain polyunsaturated fatty acids (PUFA) are bound to PLs (10). Moreover, plasma PC is a sensitive biomarker for n-3 PUFA intake in young women (11). Furthermore, DHA decreases in erythrocyte PL and milk FA from the last stage of pregnancy to the 6 month of lactation in Chilian mothers (12). Although, PL and FA compositions of bovine milk fat globule membranes are modifiable with linseed-supplemented diet (13), it is unclear whether the variation in milk DHA affects the profile of PLs in the human milk. We hypothesized that milk DHA affected the PL profile in the human milk in a population with elevated milk DHA content.

The objectives of this study were to characterize the profile of milk PLs and identify a possible association between milk DHA and PL concentration in the human milk among Japanese women.

## METHODS

### Participants and Milk Samples

This study was performed as a cross-sectional analysis of the Japanese Human Milk Study cohort in the early phase of a longitudinal study (9,14). Healthy mothers and their infants were recruited from medical institutions across Japan from February 2015 to June 2017. Women were included in the study if they: (1) were healthy and lactating, (2) of Japanese ethnicity and domicile, (3) had singleton infants who were healthy at 0–6 months postpartum, and (4) were willing to collect milk samples and complete a dietary survey according to telephonically delivered and documented instructions. The milk samples for PL analysis were collected and classified as per the highest (high milk DHA group) and lowest (low milk DHA group) concentrations of milk DHA in accordance with previous studies (See Text, Supplemental Digital Content 1, http://links.lww.com/PG9/A22, which explains the sample collection in further detail).

### Questionnaire for Maternal Diet, Sociodemographic, Anthropometric, and Birth-Related Characteristics

Maternal diet during lactation was estimated using a food frequency questionnaire (9). Briefly, all study participants completed the Brief-type, self-administered, Diet History Questionnaire (BDHQ) at the time of every visit. The BDHQ is a fixed-portion questionnaire that assesses dietary intake during the previous month (15,16). For this study, we used energy-adjusted intakes (per day and 1000 kcal) for further analyses according to the BDHQ validation study (15,17). The frequency of supplement consumption was categorized as never, infrequent (1–4 days/wk), frequent (5–6 days/wk), daily, or supplemented but not recorded. A further questionnaire that included queries on both the mother and infant was administered to obtain information regarding sociodemographic (age, maternal education, and household income), anthropometric (maternal body mass index and infant birth weight and length), and birth-related environmental factors (delivery, gestation, parity, and sex) for analysis.

### Analyses of Milk Macronutrient, Energy, and Fatty Acid Composition

The compositional analyses, excluding that of PLs, are described in our previous study (9). Briefly, macronutrients and energy analyses were performed using a mid-infrared transmission spectroscopy device (Miris Human Milk Analyzer, Miris, Uppsala, Sweden) developed for in-hospital analysis of human milk. FA was analyzed with gas chromatography using an Agilent 7890B Series Gas Chromatograph system interfaced with a flame ionization detector (Agilent Technologies, Santa Clara, CA). All experimental procedures were performed in duplicates.

### PL Analysis

The total lipid was extracted using a modification of the method of Svennerholm and Fredman (18). The polar lipid was enriched using solid phase extraction (See details, Supplemental Digital Content 2, http://links.lww.com/PG9/A23, which describes the lipid extraction and solid phase extraction). Analysis of PLs was performed according to MacKenzie et al (19). The polar lipid fraction was dispersed in a sodium cholate detergent system containing internal standard (0.7 mL) and analyzed using ^31^P-NMR. Glyphosate was used as the internal standard for quantification. A detergent solution (pH 7.1) was prepared containing D_2_O (20%; for deuterium field-frequency lock capability), sodium cholate (10%, w/w), ethylenediaminetetraacetic acid (1%, w/w), and the internal standard (0.3 g/L). The polar lipid fraction was mixed with the detergent solution by vortexing, and then dispersed by ultrasonication with occasional shaking at 60 °C for up to 10 min. pH was adjusted with aqueous sodium hydroxide. The aliquot was transferred to a 5-mm NMR tube for analysis.

Quantitative NMR spectra were measured on the 2-channel Bruker Avance III 500 MHz NMR spectrometer operating at 121.5 MHz for ^31^P using WALTZ-16 inverse-gated proton decoupling for the suppression of nuclear Overhauser effect. Samples were maintained at 30 °C during the NMR measurement. The spectrometer operated at 121.5 MHz for ^31^P. A 10-μs 90 pulse was used to acquire data using a spectral width of 50 ppm (10 122 Hz) with the ^31^P transmitter offset set to 10 ppm, the ^1^H decoupler transmitter offset set to 4 ppm, and 131 072 complex data points were acquired (acquisition time 6.47 s) with a recycle delay of 6.0 s. A total of 192 scans were added after acquiring 4 dummy scans. Spectra were processed with a standard exponential apodization of 0.2 Hz before Fourier transformation, followed by manual phasing and baseline correction.

Peak resolution in the ethanolamine plasmalogen (EPLAS), PE, and SM regions of the spectrum can be poor; therefore, after analysis of intact PLs, the sample was deacylated using mild basic reagent monomethylamine (40% weight in water, 3 mL, 55 °C, 1 h). The reagent was removed under nitrogen at 60 °C, and the sample was dispersed in water and reanalyzed using ^31^P-NMR.

Chemical shifts were measured relative to the internal standard and also relative to SM. Molecular weights were calculated from average fatty acid chain lengths for dairy phospholipids (20). The observed accuracy and precision were 97%–105% and 2.2%–2.6% for major PL subclasses in the ^31^P-NMR, according to PC, PI, and PE in a soy lecithin in our previous study (19).

### Statistical Analysis

In this exploratory study, the scarcity of quantitative data on FA content in lactating women who used DHA supplements precluded a power calculation. The sample size was set at 10 subjects per group (high and low milk DHA groups), according to the estimated feasibility of analyses in this and previous studies’ subcohorts at each participating study center over a 2-year period. Descriptive statistics were used to describe the study participants. In view of the nonnormal distribution (tested graphically using the Shapiro–Wilk test), continuous variables are presented as the median and interquartile range (IQR). Differences in characteristics between supplement users and never-users were investigated using the Mann–Whitney *U* test owing to unequal variance. The statistical analyses were performed using SPSS Statistics version 26.0 (IBM Corp., Armonk, NY) and R 3.2.1 (R Foundation for Statistical Computing, Vienna, Austria). A *P* value <0.05 was considered significant.

### Ethics

The study protocol was approved by the Internal Review Board of Fukuda Clinic (approval number IRB20140621-03) and registered in the Japanese Clinical Trials Registry (reference UMIN000015494). All study procedures were performed in accordance with the guidelines of the Declaration of Helsinki of 1975 as revised in 1983. All study participants provided written informed consent at enrollment in the Japanese Human Milk Study.

## RESULTS

### Subject Characteristics and Maternal Diet

Twenty mother-infant dyads participated and were classified into high and low milk DHA populations (n = 10 per each group). Table 1 shows the characteristics of participants (See Text, Supplemental Digital Content 3, http://links.lww.com/PG9/A24, which summarizes the selection of the participants). The median of maternal intake of DHA was higher, but not significant, in the high milk DHA group than in the low milk DHA group (241.8 versus 183.0 mg/1000 kcal, *P* = 0.070). Maternal intake of grilled fish (24.93 versus 6.76 g/1000 kcal, *P* = 0.003) and fish with edible bone (3.39 versus 0.00 g/1000 kcal, *P* = 0.007) was significantly higher in the high milk DHA group than that in the low milk DHA group. Additionally, significant differences were noted regarding annual household income (*P* = 0.035; higher in the high milk DHA group) and infant age (*P* = 0.007; lower in the high milk DHA group), respectively.

**TABLE 1.** Maternal and Infant Characteristics

	All Participants	Milk DHA Concentration		
	(n = 20)	High (n = 10)	Low (n = 10)	*P*
Maternal characteristics				
Age, y	33 (29–36)	34 (29–35)	32 (29–36)	0.939
BMI, kg/m^2^				
Current	21.0 (19.3–21.8)	20.3 (19.1–21.7)	21.3 (19.6–21.9)	0.307
Prepregnancy	19.8 (18.8–21.2)	19.3 (18.2–20.4)	20.0 (19.0–22.1)	0.199
Education, n (%)				0.837
JHS/HS/Other	5 (25)	2 (20)	3 (30)	
Some college/technical	5 (25)	3 (30)	2 (20)	
4-y college/graduate degree	10 (50)	5 (50)	5 (50)	
Household income (Jpy/y), n (%)				0.035*
<1 999 999	0 (0)	0 (0)	0 (0)	
2 000 000–3 999 999	3 (15)	0 (0)	3 (30)	
4 000 000–5 999 999	7 (35)	3 (30)	4 (40)	
6 000 000–7 999 999	9 (45)	6 (60)	3 (30)	
8 000 000–9 999 999	1 (5)	1 (10)	0 (0)	
10 000 000<	0 (0)	0 (0)	0 (0)	
Maternal nutrient intake from diet				
Energy, kcal/d	1685.7 (1595.0–1912.4)	1685.7 (1591.7–1759.5)	1690.9 (1598.3–2078.3)	0.597
Fat, g/1000 kcal	33.1 (29.7–37.2)	34.5 (30.1–37.5)	32.0 (29.5–35.4)	0.326
SFA, g/1000 kcal	8.77 (7.69–11.45)	9.24 (7.86–11.41)	8.19 (7.32–11.49)	0.762
MUFA, g/1000 kcal	11.83 (10.73–12.54)	12.02 (10.73–12.55)	11.61 (10.73–12.43)	0.450
PUFA, g/1000 kcal	7.76 (7.10–8.22)	7.86 (7.69–9.04)	7.47 (6.91–8.17)	0.174
n-3 PUFA, g/1000 kcal	1.34 (1.18–1.52)	1.44 (1.34–1.53)	1.23 (1.14–1.52)	0.131
C22:6n-3 DHA, mg/1000 kcal	207.1 (183.0–269.0)	241.8 (205.3–274.4)	183.0 (149.1–224.3)	0.070
n-6 PUFA, g/1000 kcal	6.28 (6.08–6.49)	6.44 (6.18–7.51)	6.19 (6.02–6.29)	0.096
Maternal fish intake, cooking method				
Grilled, g/1000 kcal	17.46 (6.76–24.93)	24.93 (18.14–29.24)	6.76 (4.71–16.79)	0.003†
Raw, g/1000 kcal	11.88 (7.35–19.50)	11.88 (8.30–20.11)	12.05 (4.80–18.90)	0.545
Boiled, g/1000 kcal	12.68 (7.07–30.33)	13.13 (4.90–37.32)	11.40 (8.10–23.35)	0.820
Deep-fried, g/1000 kcal	6.40 (3.98–11.37)	6.33 (0.00–11.20)	6.65 (5.06–11.53)	0.519
Maternal seafood intake				
Shellfish, g/1000 kcal	4.52 (3.36–8.10)	6.06 (3.98–8.35)	3.82 (3.14–6.32)	0.226
Fish with edible bones, g/1000 kcal	2.33 (0.00–4.54)	3.39 (2.80–5.87)	0.00 (0.00–2.19)	0.007†
Canned tuna and bonito, g/1000 kcal	1.91 (0.73–2.78)	2.19 (1.83–3.49)	1.60 (0.00–1.92)	0.080
Dried fish, g/1000 kcal	5.50 (3.49–7.34)	7.11 (4.17–15.19)	3.99 (3.32–6.43)	0.112
Oily fish, g/1000 kcal	6.83 (3.82–8.22)	6.83 (3.90–8.22)	6.86 (3.14–8.22)	0.821
Oil-free fish, g/1000 kcal	4.50 (3.29–13.24)	4.39 (3.83–17.47)	4.58 (3.14–9.02)	0.940
Seaweed, g/1000 kcal	2.45 (1.40–4.51)	2.81 (1.59–6.86)	1.97 (1.09–3.05)	0.130
DHA supplement use, n (%)				0.073
Never, n (%)	14 (70)	5 (50)	9 (90)	
Infrequent (1–4 times/week), n (%)	2 (10)	1 (10)	1 (10)	
Frequent (5–6 times/wk), n (%)	1 (5)	1 (10)	0 (0)	
Daily, n (%)	2 (10)	2 (20)	0 (0)	
Birth and infant characteristics				
Cesarean delivery, n (%)	4 (20)	1 (10)	3 (30)	0.582
Gestational period, wk	40 (38–40)	40 (38–40)	39 (38–40)	0.636
Age, d	109 (70–134)	81 (45–109)	130 (111–183)	0.007†
Birth length, cm	49 (49–51)	50 (49–51)	49 (48–51)	0.934
Birth weight, g	3236 (2897–3381)	3313 (2790–3390)	3153 (3004–3360)	0.821
Sex, male, n (%)	13 (65)	7 (70)	6 (60)	1.000
Parity, n	2 (1–2)	2 (1–2)	2 (2–2)	0.161
Breastfeeding characteristics at sample collection				
Exclusive breastfeeding, n (%)	18 (90)	9 (90)	9 (90)	1.000

Data are shown as median (interquartile range) or n (%). The values for categorical variables are shown as n (%) and those for continuous variables with a skewed distribution as the median (interquartile range).

Differences in variables according to frequency of use of DHA supplementation were examined using the Mann-Whitney *U* test. Dichotomous variables were compared by Fisher’s exact test. BMI = body mass index; DHA = docosahexaenoic acid; HS = high school; JHS = junior high school; JPY = Japanese Yen; MUFA = monounsaturated fatty acids; PUFA = polyunsaturated fatty acids; SFA = saturated fatty acids.

*P < 0.05.

†*P* < 0.01.

### Milk Macronutrients and Fatty Acid Composition

The concentration, energy, and proportion of macronutrients in milk FA are listed in Table 2. The median total fat content was 2.9 and 2.5 g/100 mL in high and low milk DHA groups, respectively. The median DHA content was higher in the high milk DHA group (1.13% [IQR, 1.06%–1.22%]) than in the low milk DHA group (0.29% [IQR, 0.26%–0.34%]; *P* < 0.001). Similar trends were observed in other n-3 long-chain PUFAs, C20:5, and C22:5. The median C20:5 and C22:5 contents were higher in the high milk DHA group than that in the low milk DHA group (0.27% versus 0.08%, *P* < 0.001 for C20:5; 0.29% versus 0.15%, *P* < 0.001 for C22:5). Collectively, the total content of PUFA was higher in the high milk DHA group than in the low milk DHA group (18.84% versus 16.19%, *P* = 0.023). Furthermore, the ratio of n-6 to n-3 PUFAs was smaller in the high milk DHA group than that in the low milk DHA group (4.38 versus 7.07). In contrast, some saturated and monounsaturated FA concentrations were high in the low milk-DHA group. There were significant differences in the contents of C16:0, C20:0, and C18:1n-9 between the high and low milk DHA groups (*P* < 0.05). Conversely, the contents of C15:0 and C20:1 were high in the high milk DHA group.

**TABLE 2.** Macronutrients, Energy, and Fatty Acid Composition in Human Milk

	All Participants	Milk DHA Concentration		
	(n = 20)	High DHA (n = 10)	Low DHA (n = 10)	*P*
Macronutrients and energy				
Fat, g/100 mL	2.5 (2.1–3.6)	2.9 (2.2–3.7)	2.5 (2.1–3.1)	0.820
Crude protein, g/100 mL	0.9 (0.8–1.1)	1.0 (0.8–1.2)	0.9 (0.8–1.0)	0.321
True protein, g/100 mL	0.7 (0.6–0.9)	0.8 (0.6–1.0)	0.7 (0.6–0.8)	0.360
Carbohydrate, g/100 mL	7.4 (7.2–7.6)	7.4 (7.2–7.6)	7.4 (7.1–7.5)	0.762
Total solid, g/100 mL	11.0 (10.7–12.2)	11.1 (11.0–12.3)	10.8 (10.5–11.8)	0.289
Energy, kcal/100 mL	56 (53–67)	60 (56–68)	56 (53–62)	0.570
Fatty acid composition				
Total SFA, %	40.4 (39.4–41.8)	40.4 (39.4–42.0)	40.5 (39.4–41.8)	0.940
C6:0	0.06 (0.00–0.48)	0.03 (0.00–0.52)	0.06 (0.00–0.08)	0.937
C8:0	0.25 (0.21–0.33)	0.30 (0.21–0.34)	0.23 (0.20–0.31)	0.199
C10:0	1.33 (1.22–1.54)	1.47 (1.29–1.59)	1.24 (1.18–1.36)	0.112
C11:0	0.01 (0.01–0.01)	0.01 (0.01–0.02)	0.01 (0.01–0.01)	0.623
C12:0	5.11 (4.33–6.24)	4.95 (4.43–7.11)	5.26 (4.30–5.57)	0.762
C13:0	0.02 (0.02–0.03)	0.02 (0.02–0.03)	0.02 (0.02–0.02)	0.112
C14:0	4.94 (4.18–5.97)	5.27 (4.38–6.05)	4.94 (4.10–5.59)	0.597
C15:0	0.21 (0.19–0.28)	0.26 (0.19–0.31)	0.20 (0.18–0.22)	0.034*
C16:0	21.26 (19.57–22.13)	20.11 (18.93–21.32)	21.96 (20.30–22.97)	0.019*
C17:0	0.26 (0.24–0.30)	0.27 (0.24–0.31)	0.25 (0.23–0.27)	0.364
C18:0	5.94 (5.45–6.57)	5.90 (5.38–6.06)	6.32 (5.72–6.66)	0.364
C20:0	0.19 (0.17–0.20)	0.18 (0.16–0.20)	0.20 (0.19–0.21)	0.023*
C22:0	0.07 (0.06–0.08)	0.07 (0.06–0.08)	0.07 (0.06–0.09)	0.597
C23:0	0.07 (0.06–0.09)	0.07 (0.05–0.08)	0.08 (0.06–0.09)	0.450
Total MUFA, %	39.33 (36.32–40.80)	38.62 (35.21–39.84)	39.82 (38.59–41.51)	0.151
C14:1	0.12 (0.10–0.16)	0.12 (0.11–0.17)	0.13 (0.10–0.16)	0.940
C16:1n-7	2.11 (1.89–2.33)	2.21 (2.07–2.40)	1.99 (1.78–2.18)	0.257
C17:1	0.16 (0.15–0.19)	0.16 (0.15–0.18)	0.17 (0.15–0.20)	0.650
C18:1n-9	34.53 (31.90–35.89)	33.66 (30.98–34.88)	35.78 (34.46–36.42)	0.034*
C18:1n-7	1.76 (1.62–1.88)	1.79 (1.64–1.91)	1.74 (1.61–1.85)	0.450
C20:1	0.44 (0.36–0.49)	0.49 (0.44–0.52)	0.37 (0.36–0.44)	0.028*
C22:1n-9	0.07 (0.06–0.09)	0.09 (0.07–0.10)	0.06 (0.06–0.07)	0.001‡
Total PUFA, %	17.00 (16.01–20.12)	18.84 (16.80–21.25)	16.19 (15.41–17.21)	0.023*
Total n-6 PUFA, %	14.53 (13.68–16.80)	15.46 (13.21–17.61)	14.19 (13.85–15.13)	0.496
C18:2	13.61 (12.81–15.81)	14.35 (12.17–16.50)	13.19 (12.87–14.29)	0.496
C18:3	0.11 (0.08–0.13)	0.11 (0.07–0.13)	0.10 (0.08–0.14)	0.940
C20:2	0.24 (0.21–0.26)	0.25 (0.23–0.26)	0.21 (0.20–0.25)	0.174
C20:3	0.25 (0.22–0.30)	0.25 (0.22–0.30)	0.24 (0.22–0.30)	0.762
C20:4	0.39 (0.34–0.44)	0.41 (0.39–0.43)	0.35 (0.32–0.44)	0.112
Total n-3 PUFA, %	2.67 (2.01–3.70)	3.70 (3.12–3.87)	2.01 (1.81–2.08)	<0.001‡
C18:3	1.55 (1.33–1.78)	1.70 (1.59–2.00)	1.41 (1.32–1.50)	0.070
C20:3	0.05 (0.03–0.06)	0.05 (0.03–0.06)	0.04 (0.04–0.05)	0.404
C20:5	0.18 (0.08–0.27)	0.27 (0.24–0.42)	0.08 (0.07–0.09)	<0.001‡
C22:5	0.21 (0.15–0.29)	0.29 (0.26–0.35)	0.15 (0.13–0.17)	<0.001‡
C22:6 (DHA)	0.66 (0.29–1.13)	1.13 (1.06–1.22)	0.29 (0.26–0.34)	<0.001‡
Others, %	3.27 (2.64–3.58)	3.57 (3.24–3.92)	2.72 (2.60–3.29)	0.049*
n-6/n-3	6.17 (4.38–7.10)	4.38 (4.10–4.85)	7.10 (7.07–8.01)	<0.001‡

Data are shown as median (interquartile range). Distributions were examined using the Mann-Whitney *U* test. DHA = docosahexaenoic acid; MUFA = monounsaturated fatty acid; PUFA = polyunsaturated fatty acid; SFA = saturated fatty acid.

*P < 0.05.

†*P* < 0.01.

‡*P* < 0.001.

### Phospholipids Profile

The profile of milk PLs among the participants of the 2 groups is summarized in Table 3. Twelve PL subclasses showed 100% positive results, while lyso-PS, cardiolipin, and phosphatidylglycerol showed 25%–40% positive results (25% for lyso-PS, 35% for cardiolipin, and 40% for phosphatidylglycerol) among all participants. The median proportions and concentrations of total PL, sphingophospholipids, and glycerophospholipids were consistent between groups. Furthermore, these findings were irrespective of milk DHA contents and the amount of total PL appearing to be stable in the milk fat content. Among the subclasses in glycerophospholipids, the median proportion of choline-containing glycerophospholipids was higher in the high milk DHA group than that in the low milk DHA group (24.09% [per total PLs; IQR 23.08–26.38, min-max 20.59–26.70] versus 21.41% [IQR 20.74–22.84, min-max 19.61–23.83], *P* = 0.019), and the median proportion of PI in the high milk DHA group was lower than that in the low milk DHA group (6.62% [IQR 5.75–6.72, min-max 4.76–7.12] versus 7.63% [IQR 7.11–8.16, min-max 6.30–8.39], *P* = 0.002). For the median proportions of glycerophospholipid subclasses, PC and alkyl-acyl PC appeared to be high in the high milk DHA group than in the low milk DHA group (21.90% [IQR 18.51–23.22, min-max 17.75–24.90] versus 19.78% [IQR 18.17–20.26, min-max 16.48–21.53], *P* = 0.059 for PC; 0.60% [IQR 0.40–0.74, min-max 0.17–2.19] versus 0.33% [IQR 0.14–0.51, min-max 0.08–0.66], *P* = 0.059 for alkyl-acyl PC).

**TABLE 3.** Phospholipid Composition in Human Milk (n = 20)

	All Participants (n = 20)			High Milk DHA (n = 10)			Low Milk DHA (n = 10)			*P*		
	Pos, %	% of Total PL	mg/100 g of Milk	Pos, %	% of Total PL	mg/100 g of Milk	Pos, %	% of Total PL	mg/100 g of Milk	Pos, %	% PL	mg/100g
Total phospholipids	100	100	18.27 (14.80–24.02)	100	100	19.76 (15.30–25.77)	100	100	17.12 (14.30–21.12)	1.000	1.000	0.450
Total sphingophospholipids	100	32.69 (31.99–33.73)	5.96 (5.10–7.63)	100	32.62 (30.52-33.57)	6.29 (5.13–8.23)	100	32.69 (32.13-34.11)	5.52 (5.08–6.25)	1.000	0.597	0.364
Sphingomyelin	100	30.62 (29.86–32.15)	5.56 (4.89–7.27)	100	30.73 (29.40–31.75)	6.03 (5.02–7.63)	100	30.62 (30.14–32.54)	5.19 (4.80–6.15)	1.000	0.650	0.364
Dihydrosphingomyelin	100	1.68 (1.36–2.02)	0.35 (0.22–0.38)	100	1.59 (1.38–1.98)	0.36 (0.23–0.39)	100	1.91 (1.33–2.05)	0.33 (0.21–0.38)	1.000	0.705	0.597
Total glycerophospholipids	100	67.31 (66.27–68.01)	12.26 (9.85–16.16)	100	67.38 (66.43-69.48)	13.46 (10.17–17.47)	100	67.31 (65.89-67.87)	11.60 (9.52–14.72)	1.000	0.597	0.364
Choline-bound glycerophospholipids	100	22.96 (20.94–24.14)	4.18 (3.32–5.41)	100	24.09 (23.08–26.38)	5.03 (3.81–5.49)	100	21.41 (20.74–22.84)	3.84 (2.97–4.50)	1.000	0.019‡	0.151
Phosphatidylcholine	100	20.26 (18.30–22.02)	3.86 (2.95–4.81)	100	21.90 (18.51–23.22)	4.57 (3.44–4.83)	100	19.78 (18.17–20.26)	3.45 (2.61–4.16)	1.000	0.059	0.174
Lysophosphatidylcholine	100	1.56 (1.05–1.87)	0.25 (0.22–0.35)	100	1.56 (0.93–1.76)	0.23 (0.22–0.36)	100	1.56 (1.39–2.17)	0.27 (0.23–0.33)	1.000	0.545	0.545
Alkyl-acyl phosphatidylcholine	100	0.43 (0.30–0.62)	0.08 (0.05–0.11)	100	0.60 (0.40–0.74)	0.11 (0.08–0.16)	100	0.33 (0.14–0.51)	0.06 (0.03–0.09)	1.000	0.059	0.016‡
Choline plasmalogen	100	0.31 (0.19–0.51)	0.05 (0.03–0.11)	100	0.46 (0.23–0.57)	0.10 (0.04–0.14)	100	0.25 (0.16–0.35)	0.04 (0.03–0.07)	1.000	0.131	0.082
Phosphatidylserine	100	9.82 (9.36–10.25)	1.73 (1.43–2.40)	100	9.82 (9.44–10.23)	1.94 (1.57–2.63)	100	9.68 (9.33–10.63)	1.58 (1.37–2.15)	1.000	0.940	0.199
Lysophosphatidylserine	25	0.16 (0.13–0.19)	0.03 (0.03–0.04)	20	0.16 (0.13–0.19)	0.04 (0.03–0.04)	30	0.16 (0.14–0.33)	0.03 (0.03–0.06)	1.000	1.000	1.000
Phosphatidylinositol	100	6.89 (6.55–7.63)	1.30 (1.02–1.59)	100	6.62 (5.75–6.72)	1.33 (0.92–1.72)	100	7.63 (7.11–8.16)	1.30 (1.08–1.50)	1.000	0.002§	0.880
Ethanolamine-bound phospholipids	100	26.12 (24.05–27.17)	4.88 (3.80–6.48)	100	25.26 (24.21-26.96)	5.08 (3.70–6.85)	100	26.68 (23.57-27.21)	4.75 (3.90–4.94)	1.000	0.650	0.414
Phosphatidyl ethanolamine	100	16.29 (13.90–17.98)	2.88 (2.25–4.22)	100	16.29 (13.77–18.00)	3.14 (2.24–4.22)	100	16.56 (15.09–17.96)	2.80 (2.31–3.20)	1.000	0.821	0.762
Lysophosphatidyl ethanolamine	100	1.84 (1.06–2.24)	0.31 (0.23–0.43)	100	1.85 (1.07–2.37)	0.29 (0.23–0.42)	100	1.84 (1.05–2.12)	0.32 (0.17–0.43)	1.000	0.545	0.762
Ethanolamine plasmalogen	100	8.07 (7.34–8.74)	1.48 (1.24–1.94)	100	8.13 (7.69–8.86)	1.78 (1.17–2.12)	100	7.96 (7.09–8.62)	1.38 (1.31–1.60)	1.000	0.705	0.226
Total plasmalogen*	100	8.39 (7.73–8.99)	1.56 (1.27–2.00)	100	8.59 (7.93-9.13)	1.86 (1.21–2.27)	100	8.22 (7.53-8.86)	1.44 (1.33–1.68)	1.000	0.545	0.226
Total ether phospholipids†	100	9.00 (8.34–9.70)	1.62 (1.33–2.12)	100	9.38 (8.67–9.74)	1.99 (1.32–2.51)	100	8.62 (8.07–9.37)	1.52 (1.34–1.70)	1.000	0.082	0.174
Cardiolipin	35	0.27 (0.13–0.36)	0.05 (0.02–0.08)	30	0.41 (0.31–0.58)	0.11 (0.07–0.11)	40	0.16 (0.05–0.29)	0.03 (0.01–0.05)	1.000	0.157	0.157
Phosphatidylglycerol	40	0.33 (0.14–0.57)	0.06 (0.03–0.10)	40	0.33 (0.23–0.44)	0.07 (0.04–0.10)	40	0.36 (0.10–0.73)	0.04 (0.02–0.10)	1.000	1.000	0.564
Phosphatidic acid	100	1.38 (0.91–1.66)	0.25 (0.18–0.33)	100	1.08 (0.74–1.52)	0.20 (0.17–0.39)	100	1.57 (1.19–1.76)	0.28 (0.21–0.32)	1.000	0.131	0.545

Data are shown as median (interquartile range). Distributions and frequencies were examined using the Mann–Whitney *U* test and Fisher’s exact test. DHA = docosahexaenoic acid; PL = phospholipids.

*Choline plasmalogen and ethanolamine plasmalogen.

†Choline plasmalogen, alkyl-acyl plasmalogen, and ethanolamine plasmalogen.

‡*P* < 0.05.

§*P* < 0.01.

## DISCUSSION

NMR is a nondestructive technique and is advantageous for valuable samples and nontargeted analysis with high specificity and selectivity (2,21). Compared with liquid-chromatography and mass spectrometry, ^31^P-NMR spectroscopy is a fast and accurate quantitation method for multiple PL classes with a single quantitative internal standard (22). To the best of our knowledge, this study is the first to conduct PL profiling analysis based on milk DHA levels, using ^31^P-NMR in the human milk from Japanese women.

The total proportions of major phospholipids for the high and low milk DHA groups (Table 3) were consistent with previous data (19.0%–37.7%, 29.0%–43.3%, 8.6%–28.0%, 5.2%–10.1%, and 3.7%–16.7% for the mean levels of PC, SM, PE, PS, and PI in the mature milk) (2). Overall, the general composition of major PL subclasses was concordant with previous reports, although compositions may vary among studies. For each PL subclass, lyso-PS, cardiolipin, and phosphatidylglycerol were only partly detected in this study. Available information on the proportion and concentration of phosphatidylglycerol in the human milk is limited. The concentrations of phosphatidylglycerol lies below the detection limits in the human milk by HPLC, while the detection limits are not indicated for phosphatidylglycerol (23). Although the HPLC analysis was technically different from the ^31^P-NMR in this study, the low positive rates for phosphatidylglycerol in this study could be attributed to the low levels of phosphatidylglycerol in the human milk. Moreover, human milk cardiolipin and lyso-PS have been insufficiently researched previously. Lyso-PS, cardiolipin, and phosphatidylglycerol accounted for less than 1.0% of total PL. In contrast, plasmalogens and alkyl-acyl PC were determined with a positive rate of 100%. Further spectral separation and sensitivity would be required to improve the results for the 3 subclasses.

Analysis of PL profile suggested that the median proportions of alkyl-acyl PC and PC appeared to be high in the high milk DHA group, although the difference was on the border-line significance as the *P* values for alkyl-acyl PC and PC were 0.059 as compared to those in the low DHA group (Table 3). Alkyl-acyl PC and PC partly share the biosynthetic pathway in the peroxisomes and endoplasmic reticulum (24); and in the plasma, PC is a sensitive biomarker for n-3 PUFA intake in young women (10). In contrast, DHA-PE-plasmalogen concentrations vary among women in the western population, and may be independent of maternal DHA intake (25). These findings support the hypothesis that an association does exist between milk DHA and choline-containing glycerophospholipids with respect to the sensitivities of milk DHA, and PL subclasses. Meanwhile, sphingophospholipids are independently biosynthesised from glycerophospholipids (26,27). The concentration of milk SM was constant throughout all stages of lactation (28). Collectively, these findings would be consistent with comparable profiles of SM, irrespective of the milk DHA.

A limitation of this study is the profiling comparison of lactating women with differing backgrounds. The inclusion criteria were based on the milk DHA concentrations found in our previous study (9), in which the high milk-DHA group comprised dyads with differing characteristics, including household income, seafood intake, and infant age. Maternal grilled fish intake and infant age were associated with milk DHA concentrations, corroborating the previous study. In contrast, a higher household income and maternal intake of fish with edible bone were not associated with milk DHA concentrations. Maternal education, age, and ethnicity are previously found to be associated with milk DHA levels (29). Moreover, low-income mothers may not reach the minimum recommended DHA levels (30). Thus, socioeconomic status may also be of great significance in Japanese populations. The variation in choline-containing glycerophospholipid profiles might be attributed to the different stages of lactation because of a lactational decrease in PC from the colostrum to mature milk (27). In crude univariate and multivariate linear regression models, infant age was independently associated with the proportion of choline-containing glycerophospholipids even when adjusted with the milk DHA levels and the milk DHA groups (*P* < 0.05, data not shown). However, in a Chinese cohort, PC and lyso-PC appeared to be consistent in mature milk from 0.5 to 8 months (31). In addition, the dietary intake of choline and PL might influence the milk PL profile. For the other factors differing between the groups, household income, grilled fish intake, and fish with edible bone intake were not associated with choline-containing glycerophospholipids in the crude univariate models. Due to the considerations of a nonnormal distribution and low sample number, possible confounding factors should be considered to verify our results in future researches.

For milk FA contents, the total n-3 PUFA, C20:5, and C22:5 were higher in the high milk DHA group than in the low milk DHA group. This could be attributed to high maternal seafood intake in the high milk DHA group. Since milk DHA concentrations are not associated with dietary DHA intake from the food frequency questionnaire (9), the differences in the maternal DHA intake is expected to be high in the high milk DHA group; however, this finding was not significant in our study. Meanwhile, some saturated and monounsaturated FAs showed higher contents in the low milk DHA group than in the high milk DHA group, including major milk FAs, C16:0 and C18:1n-9. Major milk FA appeared to compensate for milk n-3 PUFA according to the maternal diet.

In conclusion, choline-containing glycerophospholipid concentrations were higher in the high milk DHA group than in the low milk DHA group, while the milk PL profile in the human milk from Japanese women was comparable to previous reports. Large variations in milk DHA levels may have affected the composition of choline-containing glycerophospholipids in Japanese mothers. However, possible confounders were not excluded in our study; therefore, further investigations and intervention studies are required to determine the clinical relevance of milk PL profile in infant development.

## ACKNOWLEDGMENTS

We thank the entire Japanese Human Milk Study team who contributed to the setup and operation of the Japanese Human Milk Study. Furthermore, we acknowledge Keisuke Nojiri, Yuta Tsujimori, Tomoki Takahashi, Shunjiro Kobayashi, Dr Jun-ichi Yamamura, and Dr Taku Nakano for collecting the data on existing sociodemographic characteristics and milk macronutrients, and assisting with sample and data management. We acknowledge Toshiaki Uchida for the management of the study team; Yuzuka Shimomura, Ryota Wakui, Hiroaki Matsuura, Hiroaki Kubouchi, and Dr Makoto Shiota for collecting the pre-existing data on milk FA composition; and Maki Azakami and Tomomi Miyazono for collecting the human milk samples and for recording the participant data in the Japanese Human Milk Study.

## Supplementary Material


