# Supplementary material for: Milk Phospholipid Profiling Among Japanese Women with Differing Docosahexaenoic Acid Levels
Source: JPGN Rep. 2021 Mar 30;2(2):e058. doi: 10.1097/PG9.0000000000000058 (PMC10191534; doi:10.1097/PG9.0000000000000058)
Supplement: Supplementary file 1 [file pg9-2-e058-s001.pdf]

Supplemented digital content (SDC) 1

Participants and milk samples

The study participants were asked to collect milk samples into breast milk storage bags after breastfeeding using a breast pump once a day for 7 days. The milk storage bag and manual breast pump (Yanase Waichi, Osaka, Japan) were supplied by the study group upon registration at the study. The samples were collectively pooled and underwent biochemical analyses for PL, FA, macronutrients, and energy composition. The milk samples were stored at -80°C until analysis. At least 50 mL of the milk samples were assessed in this study for analysis without affecting the amount of milk required for the analyses performed in the Japanese Human Milk Study. If a mother had more than one milk sample available at any of the assessments performed at 2-month intervals over 6 months, the most recent eligible sample was included in the study.

Women were excluded if they were taking medications or were deemed unsuitable (e.g., if there was a recent history of microbial infection that could be transmitted via a milk sample) for participation in the study as per the opinion of the investigators.
