# Supplementary material for: Milk Phospholipid Profiling Among Japanese Women with Differing Docosahexaenoic Acid Levels
Source: JPGN Rep. 2021 Mar 30;2(2):e058. doi: 10.1097/PG9.0000000000000058 (PMC10191534; doi:10.1097/PG9.0000000000000058)
Supplement: Supplementary file 2 [file pg9-2-e058-s002.pdf]

Supplemental Digital Content 2

Phospholipids composition

An 8-g milk sample in a 40 mL centrifuge tube was mixed with 16 mL of methanol and 8 mL of chloroform, sonicated for 10 min, and centrifuged at 2850 rcf for 10 minutes. The aqueous phase and pellet were further extracted once with chloroform (6 mL)/methanol (6 mL) and twice with chloroform (6 mL)/methanol (3 mL) with the use of ultrasonication. The centrifuged lipid extracts from each extraction were combined and transferred into a 100 mL centrifuge tube and washed with water (19 mL) and 10% aq. KCl (3 mL). Thereafter, they were centrifuged at 2850 rcf for 10 minutes. The lower layer was dried and constituted the crude lipid extract.

The total lipid extract was dissolved in 10 mL chloroform (HPLC grade, ThermoFisher Scientific, Waltham, MA, United States). SPE cartridges with silica gel (Sep-Pak Vac 6cc [500mg] Certified Silica Cartridges, Waters, Milford, MA, United States) were pre-conditioned with 10 mL chloroform. The sample solution was loaded, followed by a 5-mL aliquot of chloroform to wash out the sample vial. The SPE cartridge was further washed with 10 mL chloroform. Polar lipids were eluted using 4 mL methanol (Merck, Prepsolv) followed by 4 mL chloroform/methanol/water (3:5:2 by volume). The polar lipid fractions were evaporated until dry under a stream of nitrogen and then in vacuo following which they were weighed.
