# Supplementary material for: Milk Phospholipid Profiling Among Japanese Women with Differing Docosahexaenoic Acid Levels
Source: JPGN Rep. 2021 Mar 30;2(2):e058. doi: 10.1097/PG9.0000000000000058 (PMC10191534; doi:10.1097/PG9.0000000000000058)

Supplemental Digital Content 3

Supplemental Figure 1: Participant disposition and flow through the study.

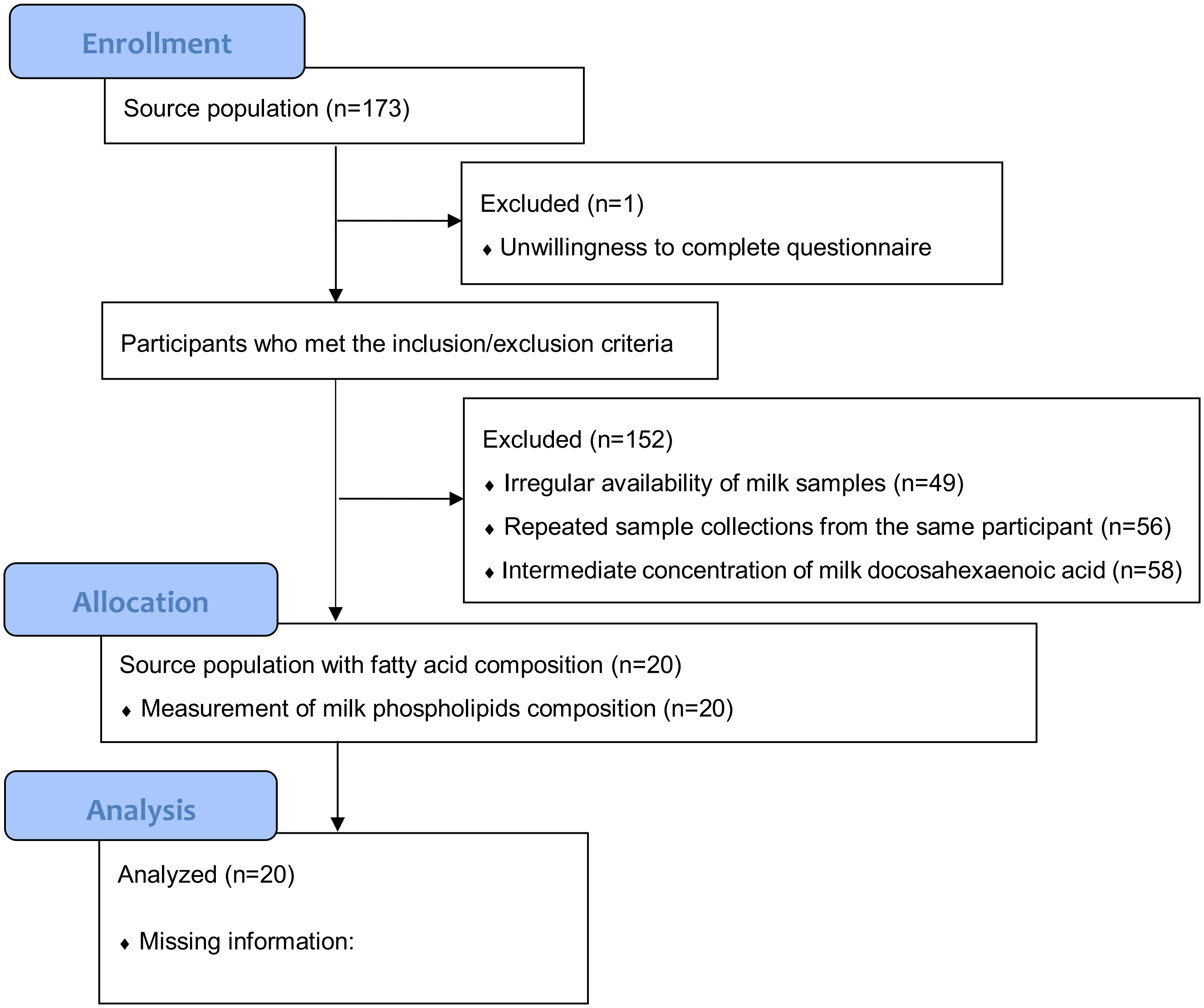

Supplement: Supplementary file 3 [file pg9-2-e058-s003.pdf]
